# Establishment and maturation of gut microbiota in White King pigeon squabs: role of pigeon milk

**DOI:** 10.3389/fmicb.2024.1481529

**Published:** 2025-01-14

**Authors:** Xiaoqin Xu, Zihan Wang, Yi Jian, Long Zhang, Caiquan Zhou, Li Liu, Hui Liu

**Affiliations:** ^1^Key Laboratory of Southwest China Wildlife Resources Conservation (Ministry of Education), China West Normal University, Nanchong, China; ^2^Institute of Ecology, China West Normal University, Nanchong, China; ^3^College of Life Sciences, China West Normal University, Nanchong, China; ^4^Agricultural Technique Promotion Station of Nanchong, Nanchong, China; ^5^Yingshan Fucheng Meat Pigeon Breeding Professional Cooperative, Nanchong, China

**Keywords:** 16S rRNA sequencing, microbiota, pigeon milk, feces, shared microbes

## Abstract

**Background:**

Pigeons are significant economic animals in China; however, research regarding the establishment and influencing factors of gut microbiota in squabs remains limited. Understanding how the gut microbiota develops in pigeons, particularly in relation to pigeon milk, is importance in pigeon production. This study aims to elucidate the establishment characteristics of the gut microbiota in White King pigeon squabs and explore the role of pigeon milk in this process.

**Methods:**

This study employed 16S rRNA sequencing technology to investigate the dynamics of microbial composition in feces and pigeon milk at various growth stages of White King pigeon. Functional prediction analysis was performed to assess the metabolic pathways involved, and correlation analysis was used to explore the relationships between microbial communities in different sample types.

**Results:**

The findings revealed a diverse microbiome present in the meconium of newborn pigeons, with a microbial composition that significantly differed from that of other feces groups. In contrast, the microbial composition of feces (FN) from pigeons aged 7 to 21 days exhibited less variability. At the phylum level, the predominant microbial taxa identified in the feces of FN were Firmicutes, Actinobacteriota, and Proteobacteria. At the genus level, the main dominant bacterial groups included *Lactobacillus*, *Limosilactobacillus*, and *Turicibacter*. Functional prediction analysis indicated that the gut microbiota of pigeons primarily participate in metabolic pathways related to carbohydrates, amino acids, lipids, cofactors, and vitamins. Furthermore, the dominant bacteria found in pigeon milk (MN) were identified as probiotics, including *Limosilactobacillus*, *Ligilactobacillus*, *Lactobacillus*, *Bifidobacterium*, and *Aeriscardovia*, which collectively accounted for over 90% of the total abundance. Correlation analysis of the abundance of shared microbes revealed that the association between meconium and feces at the other stages was extremely low. In contrast, the correlation between colostrum and feces at the post-feeding stage were found to be the highest.

**Conclusion:**

This study indicates that prenatal colonization occurs in White King pigeons. Notably, within the first week after birth, the gut microbial composition of young pigeons becomes stable. Furthermore, the colostrum serves as the most significant driver for the establishment of intestinal microbiota in squab post-birth. The findings of this study suggest that microorganisms can be added to artificial pigeon milk based on the predominant microbial composition of colostrum. This approach could facilitate the establishment of gut microbiota in young pigeons, thereby promoting their growth and development and providing production benefits.

## 1 Introduction

The gut microbiota of poultry is vast, complex, and diverse. Apajalahti et al. found that approximately 10^9^ to 10^11^ microorganisms, belonging to 140 genera and 640 species, reside solely in the chicken cecal feces (Apajalahti et al., 2004). These gut microbes play a crucial role in the host’s intestinal energy and material metabolism, extracting energy from indigestible carbohydrate feed and producing essential energy substrates, and directly absorbable nutrients, such as short-chain fatty acids, lactic acid, vitamins, and amino acids (Tannock and Liu, 2020; Vrieze et al., 2010). Additionally, they significantly contribute to the regulation of the immune system by stimulating immune cell proliferation, enhancing humoral immunity, and inhibiting the growth and reproduction of harmful bacteria (Dogi et al., 2008; Li et al., 2024; Samli et al., 2010). The early colonization and establishment of gut microbiota are closely linked to host growth and development, profoundly influencing later growth and production (Aimeric et al., 2018; Rubio, 2019). Furthermore, the effectiveness of subsequent nutritional interventions is often constrained by the already-established gut microbiota homeostasis of the host (Kogut, 2019; Rubio, 2019). Therefore, research on microbial colonization and community establishment is of utmost importance in poultry production.

Early studies have indicated that the establishment of gut microbiota in birds commences postnatally, as the digestive tract of healthy newborn birds is sterile, and microbiota colonize the intestine through external contact. However, numerous recent studies have revealed that prenatal colonization of gut microbiota also occurs in birds, with transmission occurring through various routes, including the environment, eggshell, amniotic fluid, or other mechanisms before birth (Dai et al., 2021; Dietz et al., 2019; Fisayo et al., 2020), with significant changes in the microbiota occurring shortly after birth, particularly during the first few days (Jinmei et al., 2017). In broiler chickens, the period from 0 to 4 days is a rapid colonization phase for the microbiota, and after 10 days, the microbial growth rate slows down, and the microbiota structure tends to stabilize (Jurburg et al., 2019; Ranjitkar et al., 2016). The gut microbiota of laying hens matures relatively slowly, achieving stability during the peak or later stages of egg production (Videnska et al., 2014). Research on three Arctic waterbirds has showed that the period from 0 to 2 days is marked by rapid colonization and growth of gut microbiota, with the gut microbiota structure stabilizing by the third day (Grond et al., 2017). Therefore, there are considerable differences in the establishment of gut microbiota among different species and between various breeds of the same species. However, while much is known about the microbiota of precocial birds, less is understood about the early establishment and development of microbial communities in altricial birds, which are born in a more underdeveloped state and rely heavily on parental care. Pigeons, as altricial birds, offer a unique opportunity to investigate the dynamics of gut microbiota in early life stage. As the fourth major poultry breed in China, pigeons are significant economic animals due to their high production efficiency and minimal environmental pollution. White King pigeons, a dual-purpose breed for both meat and eggs, are widely reared; however, research on the colonization and establishment of gut microbiota in White King pigeons remains scarce.

The establishment of the gut microbiota in birds is a dynamically evolving process influenced by numerous factors, with the impact of food-source microbiota being particularly significant, in addition to genetic factors (Aimeric et al., 2018; Teyssier et al., 2018; Flint et al., 2015). Pigeons, as altricial birds, are unable to feed independently at birth and rely on pigeon milk regurgitated by their parents for nourishment. After approximately 21 days, they are capable of foraging independently, although they still require parental feeding. This pigeon milk, which is rich in proteins, fatty acids, and minerals (Xie et al., 2019), as well as a diverse array of microbiota (Ding et al., 2020; Shetty et al., 1990), serves as the sole food source for newborn squabs. Consequently, the microbiota present in pigeon milk can directly enter the gastrointestinal tract, thereby influencing the establishment of the early gut microbiota in pigeons.

16S rRNA sequencing is widely used in the analysis of bacterial community composition due to its high sensitivity, cost-effectiveness, and other advantages (Schriefer et al., 2018). Hence, this study employs 16S rRNA sequencing to investigate the dynamics of microbial composition in feces and pigeon milk at various growth stages of White King pigeon squabs, with a particular focus on analyzing their functional roles. By conducting correlation analyses, we seek to elucidate the contributions of microbial communities in pigeon milk and prenatal colonization to the gut microbial colonization and flora establishment in squabs across these developmental stages.

## 2 Materials and methods

### 2.1 Animal model and sampling

Pigeon milk samples from White King pigeons were collected in Nanchong, Sichuan province, China. The experimental parent pigeons, approximately 2 years old, and at the peak of their egg production, maintained a stable egg-laying cycle. The pigeons were housed under uniform conditions: each male-female pair was kept in a cage and fed three times daily, with access to free water and natural light.

Squabs are entirely dependent on pigeon milk produced by their parents for survival during the first 21 days after hatching and cannot feed independently during this period. This period provides an optimal window for investigating the establishment of early gut microbiota in squabs and the impact of pigeon milk on its colonization. Therefore, this study collected samples at four time points during this stage for analysis. Fecal samples from the squabs were collected at four stages: day 0, day 7, day 14, and day 21, designated as F0, F7, F14, and F21, respectively, with six replicates for each group. Notably, the F0 samples presented the first feces from newly hatched squabs that had not yet consumed any food, and were therefore considered as meconium. Meconium differs from other fecal samples as it primarily consists of substances ingested by the squab from the egg before external feeding begins. The samples (FN) from F7, F14, and F21 were obtained from the same cohort of squabs. To minimize the interference of environmental microbiota, all samples in this study were carefully extracted from the cloaca by gently pressing after massaging the abdomen of squabs. These samples were immediately collected in 2 mL sterile cryovials.

Pigeon milk samples were also taken from four stages of the parent pigeon’s lactation period: day 0, day 7, day 14, and day 21, and were named as M0, M7, M14, and M21 respectively, with six replicates for each group. All pigeon milk samples are denoted as MN. To reduce slaughter, the pigeon milk was not collected directly from the parent pigeons after execution, but rather from the aforementioned squabs. As soon as the squabs were fed by their parents, the pigeon milk was expelled from the squabs’ crops into their mouths and then collected for immediate freezing in liquid nitrogen for microbial DNA extraction. Among these, the samples in group M0 were collected immediately after the newly hatched squabs were first fed by their parents, categorizing them as the pigeon’s colostrum.

### 2.2 DNA extraction and 16S rRNA gene amplicon sequencing

Microbial genomic DNA was extracted from frozen fecal and milk samples using the OMEGA Soil DNA Kit (D5625-01) (Omega Bio-Tek, Norcross, GA, United States), in accordance with the manufacturer’s instructions. The V3–V4 hypervariable region of the 16S rRNA genes was PCR amplified from the extracted microbial genomic DNA utilizing the forward primer 338F (5′-ACTCCTACG GGAGGCAGCA-3′) and the reverse primer 806R (5′-GGACTACHVG GGTWTCTAAT-3′). All PCR reactions were conducted in a total volume of 25 μL, comprising 5 μL of 5× buffer, 0.25 μL of Fast Pfu DNA Polymerase (5 U/μL), 2 μL (2.5 mM) of dNTPs, 1 μL (10 μM) of each forward and reverse primer, 1 μL of template DNA, and 14.75 μL of ddH_2_O. The thermal cycling protocol included an initial denaturation step at 98°C for 5 min, followed by 25 cycles of denaturation at 98°C for 30 s, annealing at 53°C for 30 s, and extension at 72°C for 45 s, concluding with a final extension at 72°C for 5 min. After purification and individual quantification, the amplicons were pooled in equal amounts, and paired-end 2 × 250 bp sequencing was performed using the Illumina NovaSeq platform.

### 2.3 Sequence analysis

The sequencing data was analyzed using QIIME 2 software (Bolyen et al., 2018), employing the DADA2 method (Callahan et al., 2016) and utilizing the Silva 138.1 database (Quast et al., 2012) for a comprehensive evaluation. After quality filtering, denoising, merging and chimera removal, the raw data generated representative non-singleton amplicon sequence variants (ASVs). Taxonomy was assigned to ASVs using the classify-sklearn naïve Bayes taxonomy classifier in feature-classifier plugin (Bokulich et al., 2018) against the database. Alpha and beta diversity metrics were estimated using the diversity plugin. Microbial functions were predicted by PICRUSt2 (Phylogenetic Investigation of Communities by Reconstruction of Unobserved States) (Douglas et al., 2020) upon KEGG^1^ databases.

### 2.4 Statistical analysis

Data analysis was conducted using IBM SPSS Version 19. Analysis of variance (ANOVA) was used to examine differences between groups, while a permutation test was applied to assess the significance of these differences. Pearson’s correlation coefficient was used for the correlation analysis, and a two-tailed test was performed to evaluate the statistical significance of the correlations.

## 3 Results

### 3.1 The sequencing data

**Figure 1:**
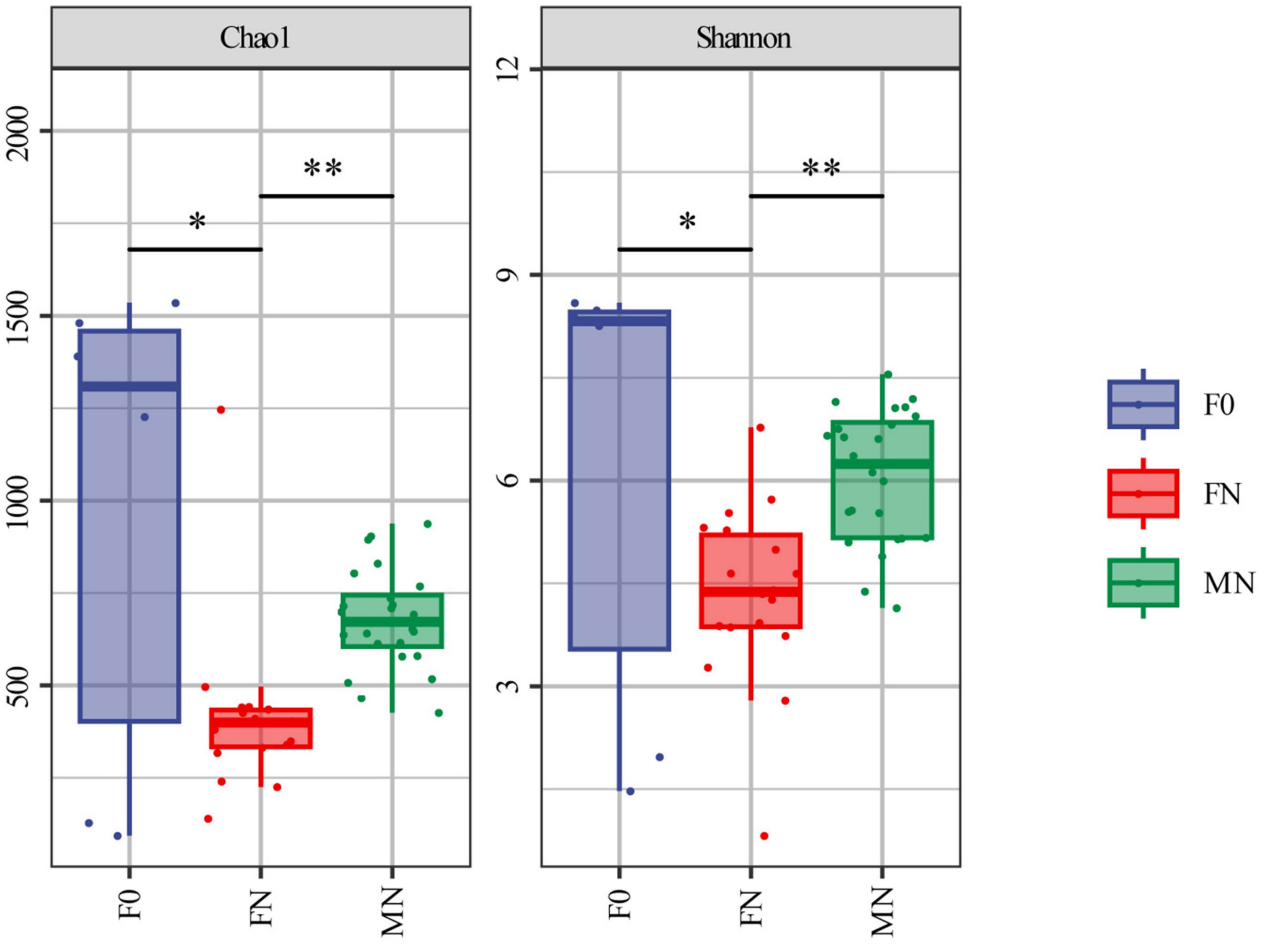
Alpha diversity analysis by Chao1 and Shannon index. ^*^The difference is significant (0.01 ≤ *p* < 0.05). ^**^The difference is extremely significant (*p* < 0.01). Error bars show standard errors.

Following quality control, a total of 3,236,401 valid sequences NCBI accession number: PRJNA1146154 were obtained from 48 samples through 16S rRNA gene sequencing, yielding an average of 67,425 sequences per sample. The average length of the clean tags was 425 bp. After rigorous clustering, we identified a total of 11,646 amplicon sequence variants (ASVs). Among these, we annotated 34 phyla, 82 classes, 182 orders, 300 families, and 612 genera in meconium. Similarly, analysis of pigeon fecal samples from FN revealed 26 phyla, 51 classes, 122 orders, 204 families, and 392 genera. Furthermore, we annotated 21 phyla, 35 classes, 70 orders, 125 families, and 234 genera in pigeon milk. The analysis of alpha diversity indicated that both the Chao1 and Shannon indices of the microbiota in meconium (F0) and pigeon milk (MN) were significantly higher than those observed in FN (Figure 1). This finding suggests a relatively high richness and diversity of bacteria in the first feces and pigeon milk.

### 3.2 Fecal microbiota dynamics during squab development

**Figure 2:**
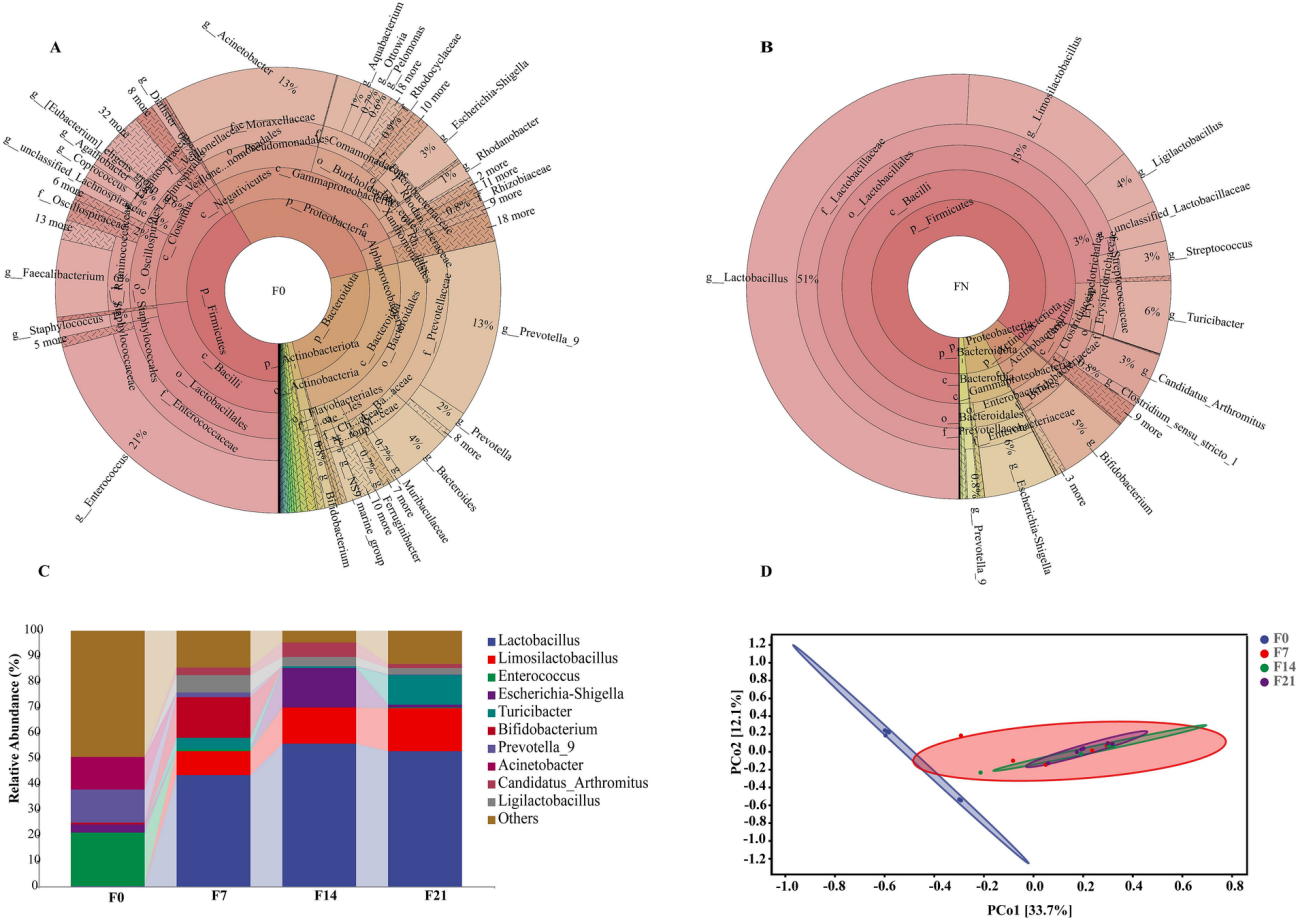
The microbiota composition and structure in pigeon feces. **(A)** The distribution of the microbiota for F0. **(B)** The distribution of the microbiota for FN. **(C)** Relative abundances of microbial communities at the genus level. **(D)** PCoA plot of microbial communities from feces with 95% confidence ellipses according to the Bray–Curtis phylogenetic distance metric.

The taxonomic composition of species at multiple taxonomic levels within feces is illustrated in Figures 2A,B. In the feces of F0, the microbial phyla with the highest abundance were Firmicutes, Proteobacteria, and Bacteroidota, while at the genus level, the most abundant genera are *Enterococcus*, *Prevotella_9*, and *Acinetobacter*. The Firmicutes phylum constituted the most significant proportion, followed by Actinobacteriota, Proteobacteria, and Bacteroidota in the feces of FN. Correspondingly, the dominant microbial genera included *Lactobacillus*, *Limosilactobacillus*, *Turicibacter*, *Escherichia-Shigella*, *Bifidobacterium*, and *Ligilactobacillus*.

The taxonomic composition analysis at the genus level revealed dynamic alterations in these microbes throughout different developmental stages (F0, F7, F14, and F21), as shown in Figure 2C. *Enterococcus* (20.82%), *Prevotella_9* (12.86%), and *Acinetobacter* (12.70%) were the primary microbiota in F0; however, their relative abundance declined rapidly, becoming extremely low in F7, F14, and F21. Conversely, the prominent probiotics, *Lactobacillus* and *Limosilactobacillus*, were present in minimal quantities in F0, accounting for 0.20 and 0.11%, respectively, but their levels increased rapidly, reaching approximately 70% in F14 and F21. Additionally, the probiotic *Ligilactobacillus* exhibited a similar trend, and these three probiotics maintained a stable presence in the later three stages. Notably, *Bifidobacterium* had a high content only in F7, accounting for 15.76%, while its levels were extremely low in other stages. *Escherichia-Shigella* was predominantly present in F14, accounting for 15.37%, but its abundance was lower in other groups, specifically 3.21% in F0, 0.14% in F7, and 1.14% in F21. *Turicibacter* had minimal presence in F0, but its levels increased gradually, peaking at 11.51% in F21. The analysis of inter-group differences (Supplementary Table S1) revealed significant discrepancies in the microbial composition of F0 compared to other stages. In contrast, the fecal microbiota compositions from 1 week to 4 weeks of age exhibited similarities, with no notable differences observed. Principal coordinate analysis (PCoA) (Figure 2D) based on Bray–Curtis distances calculated from the adjusted ASV, indicated a marked separation of F0 samples from those of other stages, while samples from the latter clustered together. This suggests a distinct microbial community in meconium compared to subsequent stages, corroborating the findings from the inter-group difference analysis.

**Figure 3:**
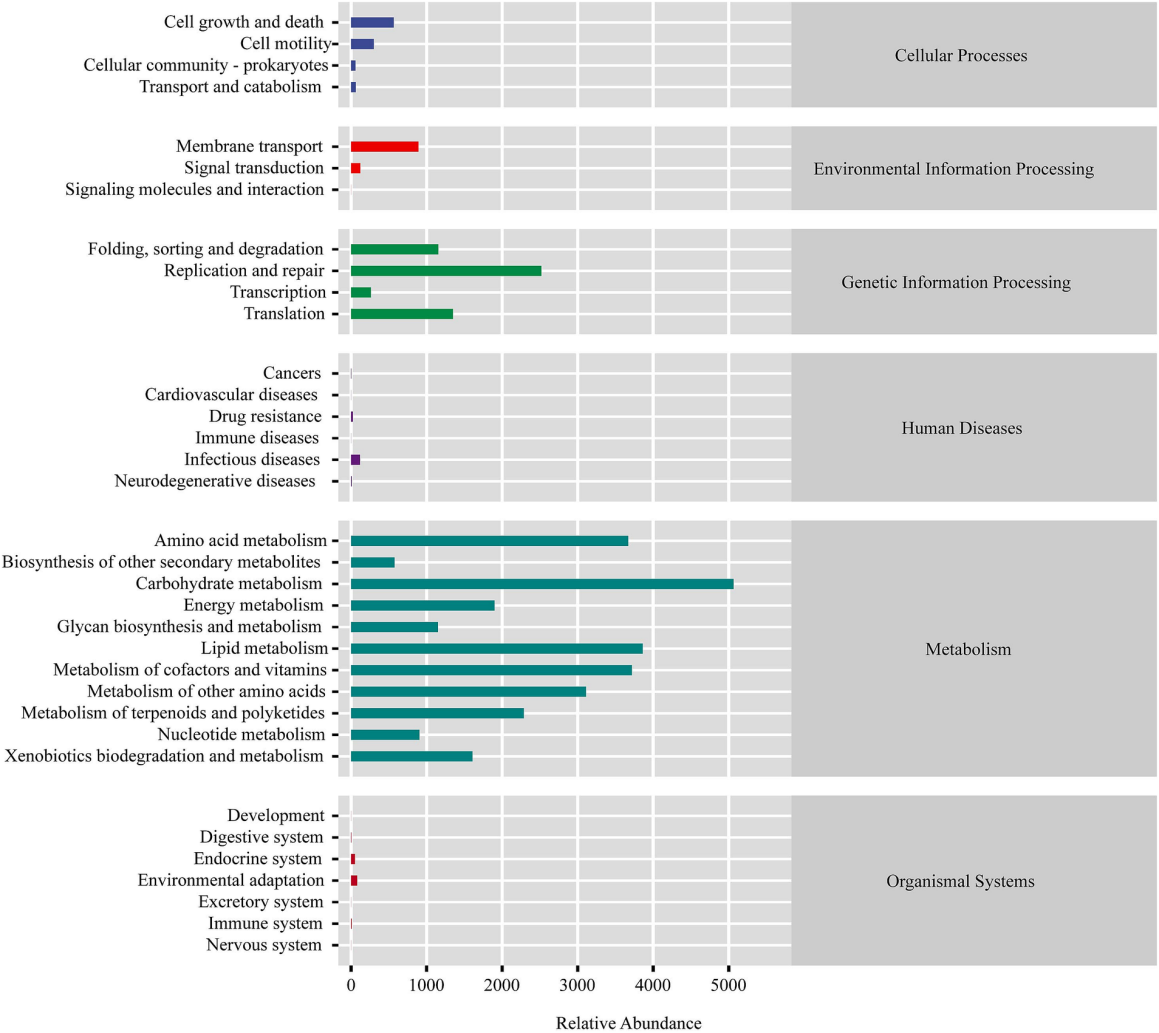
KEGG pathway enrichment analysis of feces microbiota from FN.

Predicted microbial functions (Figure 3) demonstrated that the squab gut microbiota plays a crucial role in the metabolism of various substances, including carbohydrates, amino acids, lipids, cofactors, and vitamins. Additionally, these microbiota are involved in genetic information processing, which encompasses replication and repair, folding, sorting, degradation, and translation. These metabolic pathways are essential for supporting the nutritional requirements and growth of squabs, particularly during early developmental stages. The microbial taxa contributing to these functions could play a significant role in shaping the gut ecosystem and promoting optimal growth.

### 3.3 The pigeon milk microbial characteristics

**Figure 4:**
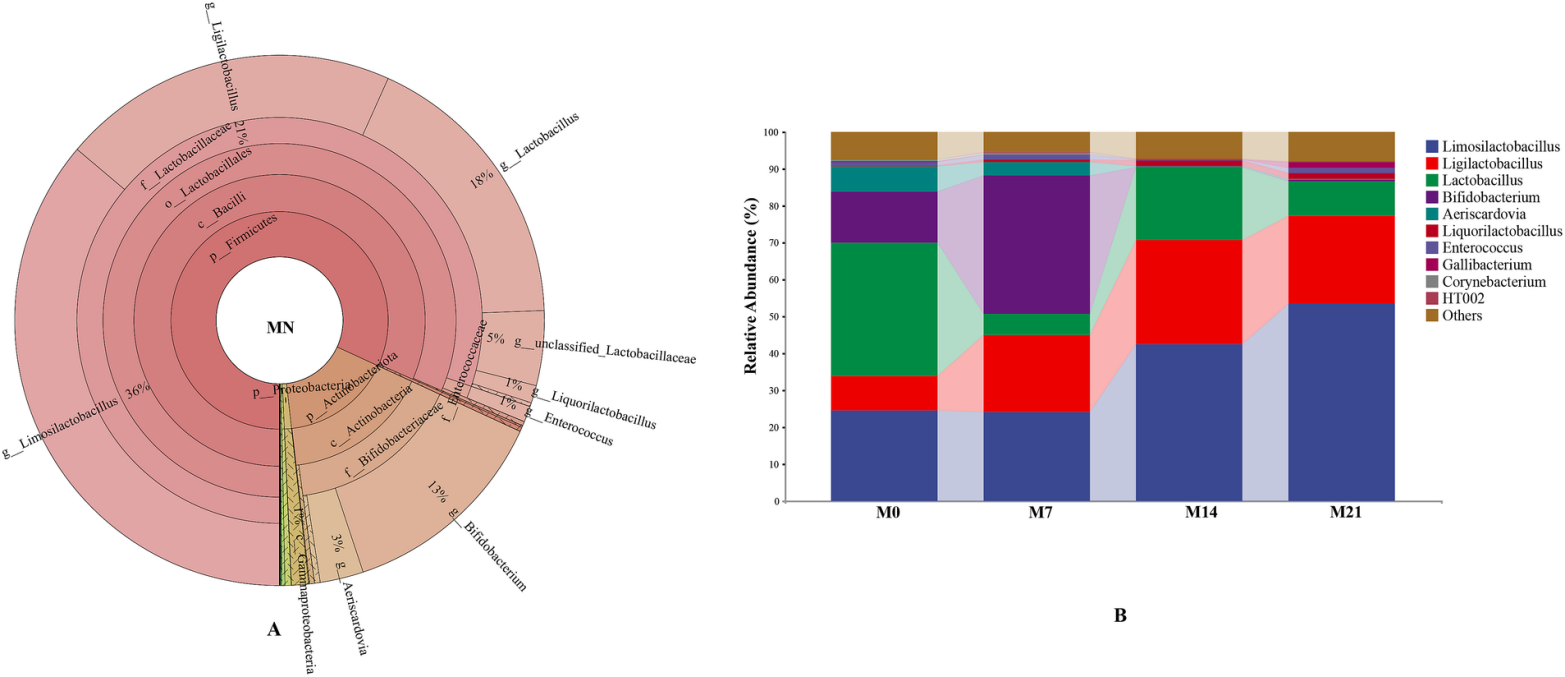
The microbiota composition in pigeon milk. **(A)** The distribution of the microbiota for MN. **(B)** Relative abundances of microbial communities at the genus level.

The predominant phylum in pigeon milk was Firmicutes, accounting for 81.86% of the relative abundance, followed by Actinobacteria (16.39%), Proteobacteria (1.06%), and Bacteroidetes (0.37%). The dominant microbial genera included *Limosilactobacillus* (36.15%), *Ligilactobacillus* (20.58%), *Lactobacillus* (17.68%), *Bifidobacterium* (13.02%), and *Aeriscardovia* (2.65%) (Figure 4A), all of which were classified as probiotics, collectively exceeding 90% in total abundance. At the genus level, there was notable variation in microbial composition across different developmental stages (M0, M7, M14, and M21) (Figure 4B). *Limosilactobacillus* and *Ligilactobacillus* emerged as key genera, exhibiting a significant upward trend, particularly in the later stages. Specifically, the abundance of *Limosilactobacillus* increased substantially from approximately 24.55% in the initial stage to 42.54% in M14 and further to 53.34% in M21. Similarly, *Ligilactobacillus* showed a marked increase from 9.24% in M0 to 23.98% in M21. In contrast, *Bifidobacterium* initially rose from 14.02% in M0 to 37.51% in M7, but then experienced a steep decline to 0.14% in M14 and 0.43% in M21. The patterns observed for *Aeriscardovia* and *Lactobacillus* were notably different, with high abundance in the early stage followed by a gradual decrease. Inter-group difference analysis (Supplementary Table S2) indicated significant differences between M0 and M7 compared with the other groups. PCoA visually supported these findings (Supplementary Figure S1).

We predicted the microbial functions in pigeon milk and discovered that the milk microbiota of pigeons shares similar functionalities with the squab gut microbiota, primarily involved in metabolic pathways (Supplementary Figure S2).

### 3.4 Effects of pigeon milk and meconium microbes on gut microbiota establishment in squabs

To investigate whether pigeon milk and meconium microbiota are transmitted to the gut of squabs, we conducted a comparative analysis at the genus level of microbiota in pigeon milk and meconium, comparing these with squab feces during the three post-feeding stages (Table 1). The number of shared microbes between meconium and squab feces gradually decreased from 281 to 152 over time. Correlation analysis of the abundance of shared microbes revealed extremely low correlations between meconium and feces at all stages. Similarly, the number and abundance correlation of shared microbes between colostrum and squab feces exhibited a decreasing trend, with the number of shared genera diminishing from 82 to 68 and the abundance correlation declining from 0.922 to 0.883. Notably, the number and abundance correlation of shared microbes between colostrum and feces at all stages was the highest compared to other stages of pigeon milk. Despite the lower number of shared microbes between pigeon milk and feces at various stages compared to meconium, the correlation of abundance for the shared microbes across these stages consistently remained higher than that of meconium.

**Table 1:** The shared microbes analysis of meconium, pigeon milk, and squab feces.

	F0			M0			M7			M14		M21
	F7	F14	F21	F7	F14	F21	F7	F14	F21	F14	F21	F21
The number of shared microbes	281	106	152	82	62	68	75	56	54	40	38	51
Correlation coefficient (*r*)	0.028	−0.010	−0.020	0.922	0.843	0.883	0.477	0.201	0.217	0.517	0.568	0.410
*p*-value	0.639	0.917	0.808	0.000	0.000	0.000	0.000	0.137	0.115	0.001	0.000	0.003

The *r* was interpreted as follows: 0 < *r* < 1 indicates a positive correlation, and −1 < *r* < 0 indicates a negative correlation. The strength of the correlation was classified based on common definitions: 0.00 ≤ ∣*r*∣ ≤ 0.19 was considered very weak, 0.20 ≤ ∣*r*∣ ≤ 0.39 was considered weak, 0.40 ≤ ∣*r*∣ ≤ 0.59 was considered moderate, 0.60 ≤ ∣*r*∣ ≤ 0.79 was considered strong, and 0.80 ≤ ∣*r*∣ ≤ 1.0 was considered very strong. Statistical significance was assessed using *p*-values: *p*-value <0.01 indicated a highly significant correlation, 0.01 ≤ *p*-value <0.05 indicated a significant correlation, and *p*-value ≥0.05 indicated no significant correlation (Yoshii et al., 2022).

**Figure 5:**
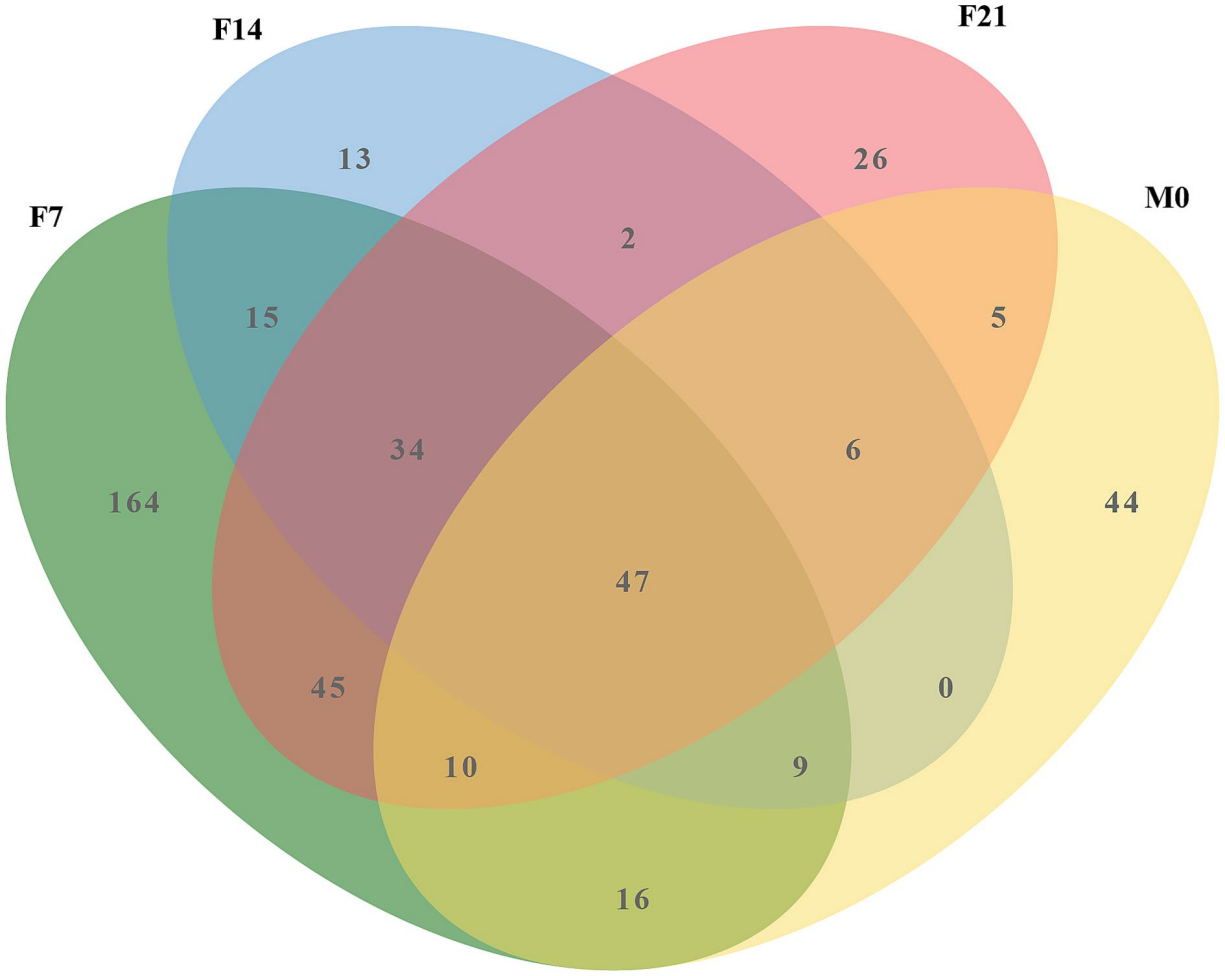
The Venn diagram of the colostrum and feces at post-feeding stages.

Venn diagram analysis (Figure 5) investigating the shared microbes between colostrum and squab feces across the three post-feeding stages unveiled a consistent presence of 47 microbial genera within the post-feeding squab gut. Notably, the majority of these genera were also detected in the prenatal gut, indicating their continuous relevance throughout developmental stages. The cumulative abundance of these 47 genera varied across different groups (F0, F7, F14, F21, M0, M7, M14, M21), with percentages reaching 74.14, 88.42, 93.22, 86.30, 97.93, 97.87, 97.55, and 95.57%, respectively (Supplementary Table S3), emphasizing their status as the predominant microbial communities in squab intestines. Among this select group, *Lactobacillus*, *Limosilactobacillus*, *Bifidobacterium*, *Ligilactobacillus*, and *Aeriscardovia* stood out for their relatively high abundances. These five probiotics are not only primary microorganisms found in colostrum but also dominate the fecal microbiota of post-feeding squabs. Their total contributions across the groups sequentially amounted to 1.125, 76.937, 73.544, 72.425, 90.653, 91.736, 90.672, and 87.204% (Supplementary Table S4), further underscoring their significance in shaping the gut microenvironment of postnatal squabs.

## 4 Discussion

Numerous studies have demonstrated that the colonization of gut microbiota begins during the embryonic period. Shortly after birth, an even greater diversity of microbiota establishes residence in the intestinal tract, gradually forming a stable and diverse microbial community that plays a crucial role in maintaining host health (Oakley et al., 2014). Consequently, we examined the microbial composition of pigeon feces across multiple growth stages, analyzed their dynamic changes, and predicted their functional roles within the gut. Diet serves as a critical driver for the establishment of gut microbiota, and pigeon milk, as the sole source of nourishment for newborn pigeons, plays a significant role in shaping their gut microbiota. Therefore, we also analyzed the microbial composition of pigeon milk at different growth stages, aiming to elucidate its role in the establishment of the gut microbiota in young pigeons.

The analysis of taxonomic composition and inter-group differences revealed significant alterations in the fecal microbiota between F0 and subsequent groups, whereas changes from F7 to F21 were relatively minor. This finding suggests that the intestines of White King pigeons are already colonized with a diverse array of microbiota prior to birth, and that these gut microbiota undergo substantial changes during the first week after hatching, followed by a period of relative stability. According to Dietz’s et al. (2019) research, prenatal colonization has been documented in rock pigeons, while Xu et al. (2022) found that the microbiota in the ileum of pigeons experiences significant variations in the first week post-hatching. These findings align with our research outcomes.

As pigeons aged, the abundance of Firmicutes in their feces gradually increased, ultimately becoming the dominant bacterial group, accounting for 97.74% at 21 days old. Firmicutes represent the most prevalent bacterial group in birds and is ubiquitous in the intestines of domestic chickens, turkeys, ducks, and other poultry (Wei et al., 2013; Yang et al., 2020). These Firmicutes species play a crucial role in facilitating digestive processes and nutrient absorption in their hosts by breaking down a diverse array of compounds, including carbohydrates, polysaccharides, sugars, and fatty acids, with the aid of digestive enzymes as catalysts (Wen et al., 2021). For example, Firmicutes produce short-chain fatty acids as fermentation byproducts, which can be directly absorbed into the host’s intestinal wall as an energy source (den Besten et al., 2013). An abundance of Firmicutes has been associated with weight gain in various animals. In domestic chickens, a positive correlation has been observed between Firmicutes abundance and both weight gain and immune function (Zhang et al., 2015). In this study, within the phylum Firmicutes, the Lactobacillaceae family exhibited a predominant relative abundance, primarily comprising *Lactobacillus* (53.03%), *Limosilactobacillus* (16.80%), and *Ligilactobacillus* (2.42%). Notably, *Limosilactobacillus* and *Ligilactobacillus* are newly classified genera that were separated from *Lactobacillus* in 2020. These genera are common gut probiotics known for maintaining gut microbiota homeostasis, enhancing immune function, promoting nutrient absorption, and alleviating inflammation to improve gut health, with widespread applications in daily life (Jiang et al., 2022; Luo et al., 2023; Rychlik, 2020; van Zyl et al., 2020; Yao et al., 2021). The high concentration of probiotics in the intestines of squab pigeons likely represents an adaptation to the high protein and fat content of pigeon milk. Furthermore, the proliferation of these probiotics may be a key factor contributing to the rapid growth and development of squab pigeons.

Diet is the primary factor shaping the gut microbiota in birds. Pigeon milk, as the exclusive food source for early-stage squabs, directly introduces its microbes into the gut via the digestive tract, significantly contributing to the establishment of the gut microbiota in these young birds. *Lactobacillus* dominated the microbial composition of pigeon colostrum and remained the predominant bacterial group in squab feces, although it was present in extremely low levels in meconium. Within just 7 days, the *Lactobacillus* content surged to 43.26%, with a gradual upward trend thereafter. Furthermore, the shared dominant bacteria (*Limosilactobacillus*, *Ligilactobacillus*, and *Bifidobacterium*) in both squab feces and pigeon milk exhibited similar trends across the four developmental stages. Specifically, *Limosilactobacillus* showed a steady increase in abundance; *Ligilactobacillus* initially rose and then declined, while *Bifidobacterium* increased consistently within the first 7 days before rapidly dropping to very low levels. Correlation analysis revealed an exceptionally high correlation in the abundance of shared microbes between colostrum and feces from all post-feeding stages. This underscores that pigeon milk not only provides essential nutrients, but also a rich microbiome, particularly abundant in probiotics, which significantly contributes to the establishment of the gut microbiota. Notably, the impact of colostrum was the most pronounced. Upon reaching the gut, dominant bacteria such as *Lactobacillus* from colostrum rapidly colonize and proliferate, inhibiting the growth of preexisting microbiota, thereby becoming the primary dominant flora within 1 week.

Although newborn pigeons were colonized by a diverse array of gut microbiota at birth, the majority of these microbes dissipated over time, leaving only a small fraction behind. The retained microbial populations, however, exhibited an extremely low correlation with the fecal microbiota of nestlings after they began feeding, suggesting that prenatally colonized microbes are transient inhabitants rather than true gut colonizers. Furthermore, the retained microbial flora is suppressed by the true colonizers that originate from pigeon milk. The disappearance of prenatal microbes is likely a dynamic process influenced by multiple factors, including environmental interactions, diet, and immune system development. These factors collectively facilitate the colonization of the gut by microbes that are better adapted to the postnatal environment (Donald and Finlay, 2023). Notably, despite their minimal impact on shaping the gut microbiota of postnatal nestlings, prenatally colonized gut microbes harbored multiple potential pathogens, including *Escherichia-Shigella*, *Klebsiella*, *Staphylococcus*, and *Pseudomonas* (Supplementary Table S5). Although these pathogens may be suppressed by dominant probiotics such as lactobacilli and their abundance remain low (van Zyl et al., 2020), they still pose a potential threat to the gut health of nestlings. Consequently, further research is warranted to explore strategies for reducing and controlling the prenatal colonization of pathogenic bacteria.

## 5 Conclusion

This study dynamically monitored the microbial composition of squab feces and pigeon milk using 16S rRNA sequencing technology. Our findings revealed a diverse microbiota present in the meconium of newborn squabs, indicating prenatal colonization in White King pigeons. Minimal variations were observed in the fecal microbial composition of squabs aged 7 to 21 days, with the dominant phyla identified as Firmicutes, Actinobacteriota, and Proteobacteria, in descending order. At the genus level, the predominant microbiota included *Lactobacillus*, *Limosilactobacillus*, and *Turicibacter*. Notably, the major bacteria present in pigeon milk were all probiotics, such as *Limosilactobacillus*, *Ligilactobacillus*, *Lactobacillus*, *Bifidobacterium*, and *Aeriscardovia*, collectively accounting for over 90% of the total microbial abundance. An analysis of the abundance correlation among shared microbes demonstrated a remarkably low correlation between meconium and feces from other developmental stages, whereas colostrum exhibited the highest correlation with feces from all post-feeding stages. This demonstrates that colostrum serves as the most crucial driving factor for the establishment of the postnatal gut microbiota in squabs. While the microbiota present in meconium appear to have a limited impact on the later development of gut microbiota, they do harbor multiple pathogenic bacteria that could potentially serve as sources of pathogens in the gut later on. To ensure the intestinal health of squabs, it is crucial to investigate the factors influencing prenatal colonization and to block potential transmission routes of pathogenic bacteria. Additionally, emphasis should be placed on disinfecting breeding eggs and incubators to minimize the introduction of pathogenic microorganisms into the embryo via the eggshell and their subsequent colonization in the gut.

## Data Availability

The original contributions presented in the study are publicly available. This data can be found here: https://www.ncbi.nlm.nih.gov, PRJNA1146154.

## Footnotes

^1^ https://www.kegg.jp/

## Supplementary material

SUPPLEMENTARY FIGURE S1 PCoA plot of microbial communities from milk with 95% confidence ellipses according to the Bray–Curtis phylogenetic distance metric.

SUPPLEMENTARY FIGURE S2 KEGG pathway enrichment analysis of milk microbiota.
