# Supplementary figures and images for: Establishment and maturation of gut microbiota in White King pigeon squabs: role of pigeon milk
Source: Front Microbiol. 2025 Jan 14;15:1481529. doi: 10.3389/fmicb.2024.1481529 (PMC11772371; doi:10.3389/fmicb.2024.1481529)

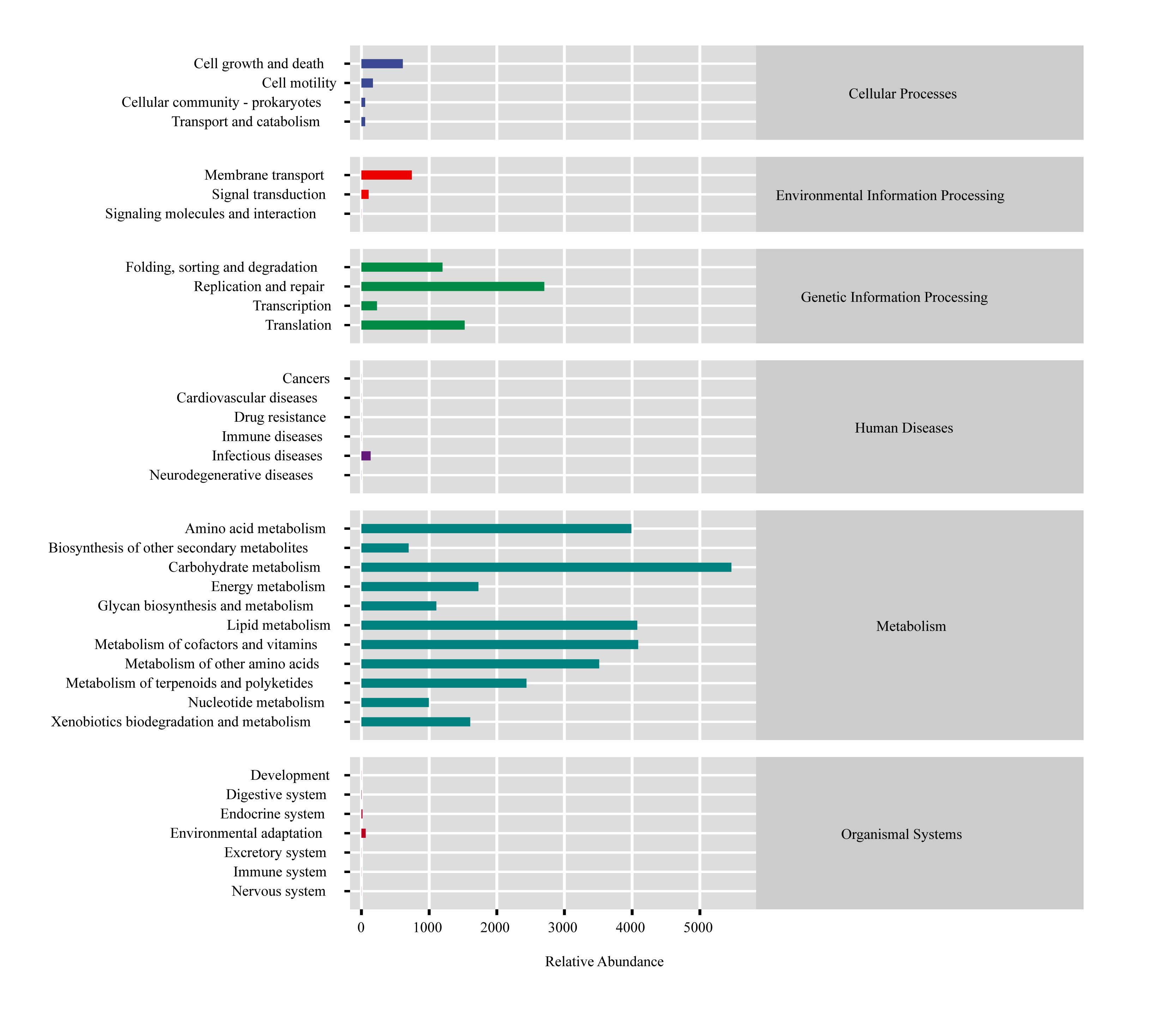

Supplement: SUPPLEMENTARY FIGURE S1 — PCoA plot of microbial communities from milk with 95% confidence ellipses according to the Bray–Curtis phylogenetic distance metric. [file Image_1.JPEG]

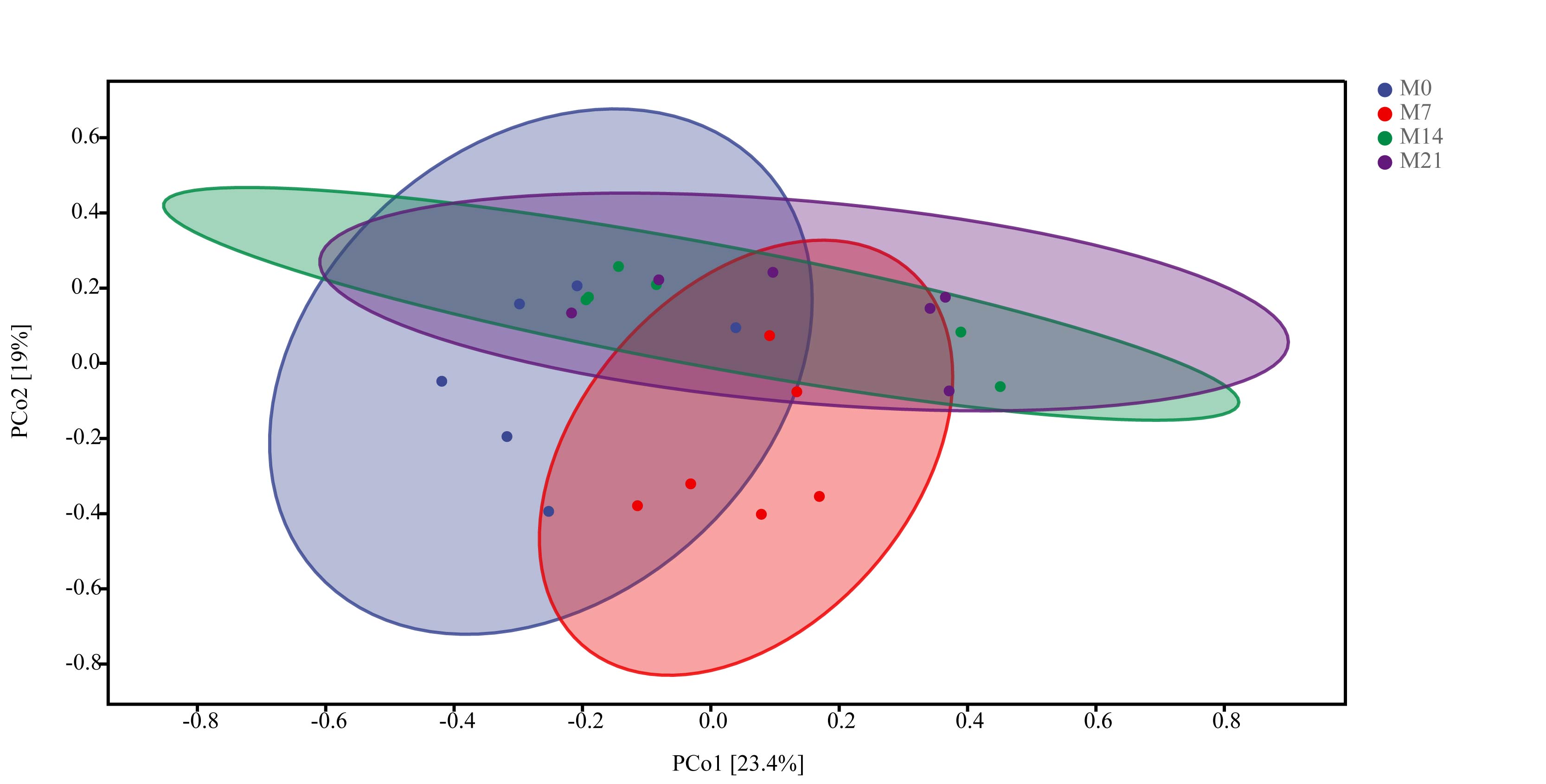

Supplement: SUPPLEMENTARY FIGURE S2 — KEGG pathway enrichment analysis of milk microbiota. [file Image_2.JPEG]
